# Plant Material as a Novel Tool in Designing and Formulating Modern Biostimulants—Analysis of Botanical Extract from *Linum usitatissimum* L.

**DOI:** 10.3390/ma14216661

**Published:** 2021-11-04

**Authors:** Sławomir Kocira, Agnieszka Szparaga, Anna Krawczuk, Petr Bartoš, Grzegorz Zaguła, Michał Plawgo, Pavel Černý

**Affiliations:** 1 Department of Machinery Exploitation and Management of Production Processes, University of Life Sciences in Lublin, Akademicka 13, 20-950 Lublin, Poland; anna.krawczuk@up.lublin.pl; 2 Department of Agrobiotechnology, Koszalin University of Technology, Racławicka 15-17, 75-620 Koszalin, Poland; agnieszka.szparaga@tu.koszalin.pl; 3 Faculty of Agriculture, University of South Bohemia in České Budějovice, 370 05 České Budějovice, Czech Republic; bartos@zf.jcu.cz (P.B.); pcerny@pf.jcu.cz (P.Č); 4 Department of Bioenergetics and Food Analysis, University of Rzeszow, Zelwerowicza 4, 35-601 Rzeszow, Poland; g_zagula@univ.rzeszow.pl; 5 ImProvia Sp. z o.o., Strefowa 13, 64-920 Piła, Poland; michal.plawgo@gmail.com; 6 Faculty of Education, University of South Bohemia, Jeronymova 10, 371 15 Ceske Budejovice, Czech Republic

**Keywords:** bioproduct, plant material, extract, flax, economy, amino acids, fatty acids, surface tension

## Abstract

Nowadays, researchers are looking into next-generation biostimulants that can be designed as a dedicated agronomic tool based on plant materials. The aim of the present study was to develop a novel biostimulating product, based on plant material in the form of linseed aqueous extracts. The scope of the research included the physicochemical characterization of the product and identification of its biostimulating potential. The study has confirmed that the plant biostimulant derived from *L. usitatissimum* can be used as a viable agronomic tool for growing soybean. The designed and produced biostimulant is rich in bioactive compounds, including amino acids, free fatty acids, carbohydrates, and micro- and macroelements. The tested biostimulant showed significantly lower values of surface tension in relation to water and a commercial biostimulant. The soybean crops responded to the application of the preparation by improvements in agronomic and morphological levels. The linseed macerates were effective in terms of soybean yields and profitability. Our findings serve as preliminary evidence for the viability of designing and developing novel biostimulants derived from plant materials. This comprehensive approach to designing and formulating novel bioproducts necessitates more extensive and targeted research to fully explain the mechanisms behind the improvements observed in the soybean cultivation.

## 1. Introduction

Over the past years, there has been increasing awareness that agriculture—as a commercial sector—has a huge impact on the environment. The increased worldwide demand for feed and food seems to have put even more pressure on this market. However, it must be stressed that the rising global demand for agricultural products should be satisfied in a way that minimizes the environmental impact of food production and consumption. The only way to achieve this goal is to develop and deploy innovative technologies to support farmers [1,2,3,4,5,6]. Given the need to mitigate the environmental damage caused by intensive agriculture, any attempt to face such challenges must include plant production and protection methods focused on stimulating the growth and development of crops, while eliminating risks to humans and the environment at the same time. With progressing climate change and growing consumer awareness with regard to plant protection and fertilization products as well as the concerns regarding Genetically Modified Organisms (GMOs), unconventional methods are needed to ensure safe and top-quality agricultural products [7,8]. Multiple research works indicate that the traditional agro-technical processes are insufficient, especially where unfavorable conditions drastically reduce yields—and thus profitability—for agricultural producers. Recently, agricultural biostimulants have received considerable attention. They are defined as products that support physiological processes in plants, while also promoting their growth and development in optimal and suboptimal conditions [9]. However, despite promising research findings, it has been noted that the biostimulant products available to farmers are limited in variety and are costly to buy and use. Moreover, the majority of popular biostimulant products are designed for use in horticulture and fruit crops. The issue is further complicated by the fact that commercial products are very rarely recommended for use in protein or legume crops (with no information on these groups of plants, timing of treatments, or recommended dosages for different methods of application) [4,10,11]. Researchers are thus looking into next-generation biostimulants that could be designed as a dedicated agronomic tool capable of meeting the demand for alternative methods based on environmentally-friendly bioactive substances that support biodiversity in agricultural ecosystems [8]. Plant extracts have been recognized as an effective group of biostimulants under new EU regulations [12]. Seeking sources for new preparations, based on the rich experience of traditional medicine and allelopathy, may thus appear to be a worthwhile line of inquiry. Based on data regarding the interaction of different plants and their antimicrobial activities, raw materials that could potentially serve as perfect candidates to produce a new biostimulant can be identified [13,14]. The constituent chemical compounds of plant biostimulants occur naturally in all higher plants, although at different concentrations and combinations [15,16]. Groups of medicinal or allelopathic plants form the basis of not only traditional medicine and pharmacology, but also organic agriculture in many cases. In recognition of their value and significance as a potential source of new agronomically-valuable compounds, initial strategies for supporting plant growth in stress conditions have already been developed [17,18]. Plant extracts tested thus far include moringa leaves, maize kernels, and licorice root [19,20,21,22,23,24,25], as well as hydrolyzed protein of plant origin [26], lemongrass [27], and garlic extract [28]. Studies conducted by Cheema et al. [29] and Farooq et al. [30] have demonstrated that aqueous extracts derived from sorghum, cabbage, sunflower, rice, wheat, barley, and moringa contain particular allelochemicals improving the growth of various crops [29]. The authors concur that the action of plant extracts and their bioactive compounds may stem from their direct and indirect effect on physiological processes in crops, validating their potential use for growth stimulation as a replacement for synthetic plant-growth regulators [30]. Plant extracts that elicit a biostimulating effect on plants can be used to improve yields [31]. Although allelopathy is considered to be an ecological phenomenon, there is still little evidence regarding its potential use in agriculture. Our previous research on application of aqueous extracts of *Artemisia absinthium* L., *Levisticum officinale* Koch., *Verbascum thapsiform* L., and *Arctium lappa* L. in three-year soybean crops showed changes in plant physiology reflected in improved biometric features. Furthermore, the treatments were found to substantially increase yields compared to the control. The key finding of the study was that the extract exerted a biostimulating effect by modifying metabolic activity and fundamental biochemical parameters of soybean [4].

In search of new species of plants with biostimulating potential, linseed was selected as a candidate due to its wide use in conventional medicine, herbal medicine, pharmacology, and cosmetology. *Linum usitatissimum* L. is a herbaceous plant species of the family Linaceae grown in almost all parts of the world. The health benefits and therapeutic action of the plant have been recognized since antiquity [32]. *Linum usitatissimum* L. has a rich chemical composition, which includes lignans, fiber, fatty acids, and amino acids [32,33,34]. *Linum usitatissimum* L. seed extracts also have a high antioxidizing potential, owing to the presence of the anti-oxidant gamma-tocopherol [35,36]. Jhala and Hall [37] have found that it is due to these properties that *Linum usitatissimum* L. is considered to be a platform or “matrix” crop for the development of novel bioproducts. Our team’s previous work showed that aqueous extracts of linseed were effective in improving germination capacity and emergence in plants and inhibited fungal and bacterial contamination of seeds of white mustard, white cabbage, yellow lupine, garden pea, fodder beet, sugar beet, beetroot, winter rape, and turnip rape, as well as spring barley grains [38,39,40,41,42]. The positive response of the seeds and seedlings to allelopathic extracts have led us to undertake research on developing a novel crop biostimulant that would not have any residual or toxic effects on the environment. The use of such a product could thus also contribute to environmental protection by helping maintain biodiversity through supporting the growth of invertebrate populations, including pollinators and other beneficial arthropods that serve as natural allies in pest control, with the preparations being designed so as not to be harmful or toxic to such species. This approach to providing effective methods for sustainable development of crop production and ecological biosystems [43,44,45,46,47] is consistent with the current EU biodiversity strategy for 2030. The criteria used by farmers when implementing new agronomic practices should also be considered—novel biostimulant products must be designed and developed with cost-effectiveness of their use in mind [4,48,49]. In this sense, a proven economic benefit is a critical success factor for the actual adoption of any bioproduct [50,51,52]. Through the lens of these factors, we conducted an investigation into the viability of using *Linum usitatissimum* L. as a source of ecological biostimulants. The aim of the present study was to develop a novel biostimulating product, based on plant material in the form of linseed aqueous extracts. The scope of the research included the physicochemical characterization of the new product and identification of its biostimulating potential and effectiveness in cultivation of Abelina variety soybean (*Glycine max* (L.) Merrill). This comprehensive approach to developing, testing, and performance profiling of the plant bioproduct will make it possible to assess its agronomic potential, and thus register and market it as a novel product.

## 2. Materials and Methods

### 2.1. Extract Production

**Figure 1:**
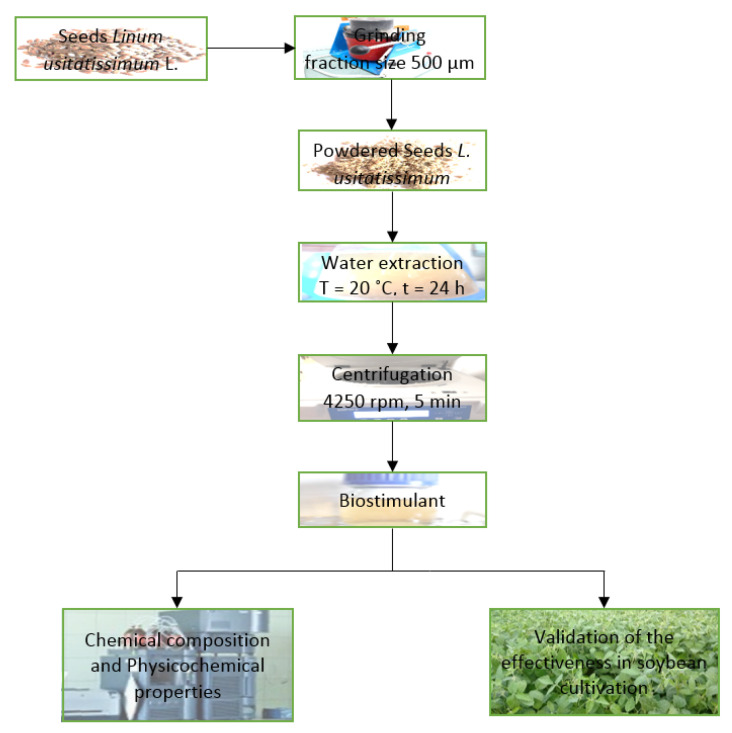
Proposed flowchart of biostimulant production and validation.

Extracts were prepared from dried, ground seeds of *L. usitatissimum* originating from biofarming (Runo, Hajnówka, Poland, PL-EKO 07 EU Organic Farming). Cold-soaked macerates from flax were prepared with the cold extraction method, i.e., 5 g of ground seeds were added to 100 mL of distilled water, and the solution was left in a dark and cold place for 24 h. Afterwards, the extracts were centrifuged at 4250 rpm for 5 min and filtered through Whatman no. 1 blotting filter paper [53]. Extracts from seeds of *L. usitatissimum* were analyzed to determine their chemical composition (Figure 1).

### 2.2. Multielemental Composition and Carbohydrate Content in Extracts from L. Usitatissimum Seeds

The samples of *L. usitatissimum* extracts were prepared in 65% extra pure nitric acid using a high pressure microwave digestion system. A total of 0.5 g of samples were placed in digestion vessels and filled up with 8 mL of acid as a reagent. The same procedure was used with a blank sample. The Ethos One microwave digestion system (Milestone, Sorisole, Italy) was used during the digestion procedure. The mineralization procedure consisted in temperature gradation and its increase to 200 °C. After all samples were filled up to 50 mL with deionized water (<0.07 µS cm^−1^). The detection threshold was higher than 0.01 mg kg^−1^ for each element (the spectrometer detection capacity is over 1 µg L^−1^). The measurements were performed by ICP-OES (Inductively Coupled Plasma Optical Emission Spectrometers, Thermo iCAP Dual 6500, Antigo, WI, USA). Each measurement was made in two planes of the plasma flame (Radial and Axial). Calibration curves were created in two concentration variants by certified Merck models. The calibration curve was built on the basis of three concentration points. For the measurement, especially of optical correctness, internal standards in the form of Y and Yb were used (Y = 2 mg L^−1^ and Yb = 5 mg L^−1^ [54].

Sugars in the extracts from *L. usitatissimum* were evaluated based on the EN 12630, 1999 standard and procedure proposed by Pereira da Costa and Conte-Junior [55] using the HPLC system.

### 2.3. Protein Amino Acids Composition in Extracts from L. Usitatissimum Seeds

The samples were filtered through a 0.22 µm syringe filter. The analysis of amino acids was performed using the amino acids analyzer AAA 400 (Ingos, Prague, Czech Republic) with a photometric detector. The amino acids were separated on a 0.37 mm × 450 mm ion exchange column (Ostion LG ANB, Ingos, Prague, Czech Republic) at 60 °C. The amino acids and proline were detected at 570 and 440 nm, respectively. The analysis was carried out for 90 min.

### 2.4. Determination of Fatty Acids in Extracts from L. Usitatissimum Seeds

The reaction mixture was acidified with 0.2 mL of 6 M HCl, and then 1 mL of deionized water was added. Released free fatty acids (FFAs) were extracted with 1 mL of hexane. After evaporation of the hexane *in vacuo*, the FFAs were methylated with 1 mL of 10% BF3 in methanol at 37 °C for 20 min. Water was added to the solution, and then fatty acid methyl esters (FAMEs) were extracted with 1 mL of hexane. FAMEs were analyzed using a Varian 450-GC gas chromatograph equipped with a flame ionization detector (FID) (Agilent Technologies, Santa Clara, CA, USA). The SelectTM Biodisel GC column, 30 mm (length) × 0.32 mm (ID) × 0.25 µm film thickness was used. Helium was used as a carrier gas with a flow rate of 1.5 mL/min. The temperature gradient of the GC oven started with an initial temperature of 100 °C, with a linear increase to 240 °C. The total run time was 20 min [56].

### 2.5. Microbiological Analyses of Non-Microbial Biostimulant

The presence of *Salmonella* spp. was estimated with the use of a simplified version of the EN ISO 6579-1:2017 method. Initial culture was carried out in buffered peptone water for 16 h at 37 °C. Selection was made in tetrathionate broth according to Müller Kauffmann (Merck, Darmstadt, Germany) after 24 h at 43 °C [57].

The horizontal method was used for the enumeration of beta-glucuronidase-positive *Escherichia coli* according to the ISO 16649-2:2001 on TBX (Tryptone Bile X-Glucuronide Agar) (BioRad, Hercules, CA, USA) after incubation at 44 °C for 24 h [3,58].

### 2.6. Physical Properties of Extracts from L. Usitatissimum Seeds

The surface tension was tested on the Drop Shape Analyzer device (DSA30 KRÜSS GmbH, Hamburg, Germany) using the pendant drop method. The pendant drop is a drop suspended from a needle in a bulk liquid or gaseous phase. The shape of the drop results from the relationship between the surface tension or interfacial tension and gravity. This method allows determining the surface tension of the liquid based on the shadow image of the pendant drop measured using drop shape analysis [59,60,61,62]. Before taking measurements, the necessary data on the diameter of the needle dispensing the measured drops (1.828 mm in external diameter) and the density of the analyzed samples (0.997 g cm^−3^) were introduced to the software that controls the operation of device. For accuracy of the measurements, the manufacturer of the device recommends that the measurements should be carried out for the largest possible volume of drops remaining on the needle. Therefore, liquid drops with the following volumes were dosed: 30 µL for water, 30 µL for the 0.5% solution of water and seaweed concentrate (Kelpak SL), and 20 µL for extracts from *L. usitatissimum* seeds. Kelpak SL was tested as a popular biostimulant used in the cultivation of soybean. The physicochemical parameters of the biostimulating extract were compared with the commercial biostimulant commonly used in agriculture. The drops were dispensed by a program control device. Then, using the input data, the program automatically determined the contours of the shape of the hanging drop and calculated the surface tension according to the Young-Laplace equation. For each analyzed liquid sample, 40 measurements were made at a temperature of 25 °C. Surface tension values were expressed in mN m^−1^ unit.

### 2.7. Plant Material and Growth Conditions

Soybean (*Glycine max* (L.) Merrill) seeds of Abelina variety originated from a three-year field experiment (2017–2019) conducted in Perespa (50°66′ N; 23°63′ E, Poland). The experiment was designed and performed in a random block system in four replications on 15 m^2^ experimental plots. Plants were grown on soil classified as Gleyic Phaeozems (pH in 1M KCl 7.3–7.4). The average level of available nutrients in 100 g of soil was as follows: 12.6–14.2 mg P_2_O, 15.2–17.1 mg K_2_O, 6.3–6.8 mg Mg, and 8.1–9.1 mg N–NO_3_ + N–NH_4_. *Triticum aestivum* L. was used as the previous crop. The seeds were sown on the 2nd of May 2017, 2018, and 2019 with 4.0 cm gaps in rows with 30 cm spacing. In each growing season, plants were treated with infusions from *L. usitatissimum*. The extract was applied in the form of double plant spraying (300 L·ha^−1^) at the BBCH 13–15 and BBCH 61 developmental stages of soybean. Combinations with plants sprayed with water (used for extract preparation) served as the control. Spraying was performed with the Pilmet 412 LUX (Unia, Grudziądz, Poland) sprayer equipped with air-induction flat fan nozzles 6MSC (working pressure 0.30 MPa). After maturation of the pods (BBCH 89), the plants were harvested. The plant height, height of the location of the first pod, number of pods, seed yield, and 1000 seed weight were determined. The dates of the application of the extracts were chosen based on results of our earlier experiments addressing the use of natural and synthetic biostimulants in soybean cultivation [52,63].

### 2.8. Evaluation of the Economic Effect

The economic effect of the *L. usitatissimum* extracts application was computed based on the value of the yield increase resulting from the use of the extracts and costs of their application in soybean cultivation [4]. Income growth (EUR·ha^−1^) resulting from the use of the extracts was calculated as a difference between the value of the yield increase and the costs of the use of the extracts. The value of the yield increase (EUR·ha^−1^) was evaluated as a product of the average price of soybean seeds in a given study year and the difference between the seed yield from the combination with the extracts and the seed yield from the control combination. The costs of the treatment with the extracts (EUR·ha^−1^) were computed as a sum of three parameters: cost of purchase of the extracts, cost of water used for the treatment, and cost of performing the treatment. The average purchase price for the seeds was determined from market offers (311.12 EUR·t^−1^). The cost of *L. usitatissimum* seeds (1.12 EUR·kg^−1^) was taken from a herbal store (Runo, Hajnówka, Poland). The cost of water was the average price of 1 m^3^ (1.50 EUR·m^−3^) in Lubelskie Province. The cost of the procedure was the average price of the plant spraying service (17.56 EUR·ha^−1^). Revenue from cultivation was calculated as the product of the yield and the price of soybeans in a given research year. The change in revenue (%) was calculated as the ratio of the revenue from the cultivation with biostimulant application to the revenue from the control.

### 2.9. Statistical Analysis

All analyses were performed in three replicates (for each year of the field experiment). The evaluation of the normal distribution of data was performed using the Shapiro–Wilk test. The results were analyzed using a one-way ANOVA. The estimation of the significance of the differences between the mean values was based on Tukey confidence intervals at a significance level of *p* < 0.05. The article presents the average results from the field experiments with the standard deviation (average ± SD). Statistica 13.3 software (TIBCO Software Inc., Palo Alto, CA, USA) was used for the analyses of the results.

## 3. Results

### 3.1. Chemical Composition of Extracts

The study showed that *L. usitatissimum* extracts contained many micro- and macroelements essential for the development of crops (Table 1). The maceration (cold extraction) produced aqueous extracts that were characterized by high proportions of calcium, potassium, magnesium, phosphorus, and sulfur. Potassium was the predominant element in the extracts, whereas phosphorus had the lowest share in the total macroelement content. Zinc and copper, which are very important macroelements for legume plant nutrition, were detected in the linseed macerates. Interestingly, the cold extraction produced plant extracts containing iron and aluminum.

The aqueous extracts were also tested for sugar content. The sucrose levels were higher compared with those of glucose. Furthermore, the macerates were found to contain 0.374 mg·mL^−1^ of fructose (Table 2). The results also revealed that the *L. usitatissimum* macerates had a varied amino acid profile. This bioproduct was rich in amino acids (Table 3). Glutamic acid, aspartic acid, and arginine were the most abundant amino acids in the aqueous extracts, whereas proline, histidine, and tyrosine had the lowest share in the total content of amino acids.

The fractions of all examined fatty acids in fat extracted from the *L. usitatissimum* extracts are presented in Table 3. The cold extracts of *L. usitatissimum* were rich in fatty acids. The macerates were found to contain heptadecanoic acid and cis-9,12,15-octadecatrienoic acid (α-linolenic acid). The analysis also showed that the macerations contained monounsaturated fatty acids, omega-3 acids, and omega-6 acids. 

The research showed that pathogens in the non-microbial biostimulant did not exceed the permissible levels (for *Salmonella* spp.: absence in 25 g and for *Escherichia coli*: 1000 in 1 g or 1 mL) specified in regulation EU 2019/1009 of the European Parliament and of the Council [64]. The results showed that no bacteria were detected in the botanical biostimulant. Thus, the evaluated non-microbial biostimulant can be considered as a safe agronomic solution and can be subjected to further investigations in the future.

### 3.2. Effect of the Application of the Extract on the Morphological Characteristics and Yields of Soybean

The application of the linseed extracts were shown to exert a strong effect on the morphological characteristics of the soybean plants (Table 4). In the three-year field study, plants treated with the novel biostimulants were taller. The evaluation of the heights of the first pod showed that the macerates had a negative influence on this parameter, leading to its lower values compared to the control. The analysis of the soybean response to the novel biostimulants confirmed their effect on the pod number per m^2^. As indicated by the test results, the yields were higher in groups treated with the linseed extract. The values of the parameter that receives most interest from farmers, i.e., the yield, improved significantly after the exogenous application of the biostimulating extracts, as the macerate-treated groups produced substantially higher soybean yields than the control crop. However, the experiments also showed that the linseed extract reduced the 1000-seed weight after application. This crop parameter was the highest in the control samples. The macerates had a negative influence on the 1000-seed weight, leading to reduction of this yield parameter by almost 2% in relation to seeds from the control cultivation. 

### 3.3. Physical Properties of the Biostimulant from L. Usitatissimum Seeds

The presented measurements of surface tension showed that the biostimulant from the *L. usitatissimum* seeds has a significantly lower average value of this parameter compared to the water and seaweed biostimulant (Kelpak SL) (Table 5).

The mean value of the surface tension of water and a mixture of water and seaweed concentrate (Kelpak SL) do not differ significantly. The measured average value of surface tension of these two samples reached a value of about 71 mN m^−1^. The extracts from the *L. usitatissimum* seeds were characterized by a surface tension value of 50.82 mN m^−1^. Compared to the other two analyzed samples, the value was approximately 29% lower. In agronomic practice, this may determine the greater efficiency of the spraying treatment, by improving the absorption of active compounds from extracts by plants, as well as increasing the adhesion of particles to the surface of plant leaves. Similar results were found for the average area of the drop surface in the bulk phase and for the average drop volume detected by the shape analysis (not the dosed volume), where there was a decrease of 23 and 30%, respectively.

### 3.4. Botanical Biostimulants versus Profits from Soybean Cultivation

**Figure 2:**
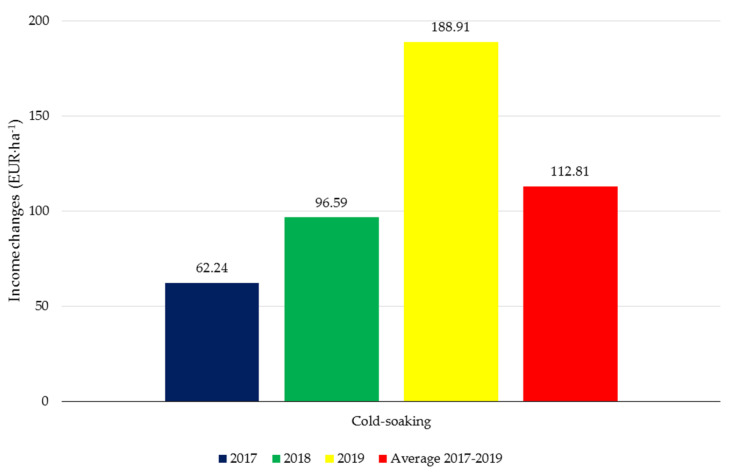
Income changes caused by the application of *L. usitatissimum* extracts in soybean cultivation. The values of the yield increase (EUR × ha^−1^) represent the difference in income from soybean cultivation with biostimulator application and control.

The application of the linseed extracts increased the profit from the cultivation of soybean (Figure 2). The use of the macerates brought high economic benefits with an average value of 112.81 EUR·ha^−1^. The results of the three-year experiment showed that the meteorological conditions in the individual growing seasons determined the soybean yield level, and thus, the recorded income. The economic benefits resulting from the use of the botanical biostimulants from flax seeds were observed in the last year of the field experiment; they amounted to 188.91 EUR·ha^−1^ after the foliar application of the cold-soaking product. 

The application of the extracts increased the revenue from the cultivation of soybean. The use of the linseed macerates resulted in higher revenue in individuals research years (15.72%, 20.95%, 30.25%). The average revenue from cultivation with biostimulant application was 22.17% higher than revenue from control cultivation.

## 4. Discussion

The results show that the biological activity of linseed aqueous extracts (macerates) is sufficient for these products to be classified as natural biostimulants. The designed and tested biostimulant complied with the requirements set out in the definition of this type of products: product able to stimulate plant nutrition processes independently of the product’s nutrient content with the sole aim of improving one or more of the following characteristics of the plant or the plant rhizosphere: (1) nutrient use efficiency, (2) tolerance to abiotic stress, (3) quality traits, or (4) availability of confined nutrients in the soil or rhizosphere [12]. The present analysis showed a varied composition of the linseed-derived plant extracts. The macerates were rich in macro- and microelements as well as sugars and amino acids. This composition suggested that they could induce a positive response in the soybean plants. Paleckiene et al. [65] have found that amino acid enrichment of crops is particularly effective if supplemented with micro- and macroelements. As suggested by Popko et al. [66], of particular importance is the pool of amino acids supplied exogenously to plants. An added source of amino acids may induce a mechanism that protects plants. Application of amino acid-containing preparations can improve the rate and direction of metabolic processes [67]. Crucially, the amino acids contained in linseed extracts play a number of essential roles in plants, e.g., chemical chelation (glutamic acid, glycine, histidine, and lysine), bolstering reproductive growth and resistance to stress (proline), cold resilience (arginine), stimulating growth (glutamic acid) and germination (aspartic acid, glutamic acid, lysine, phenylalanine, and tyrosine), regulating water balance (proline, serine), and stimulating hormone metabolism and antimicrobial resistance (alanine). Amino acids identified in plant extracts also include those that serve as precursors to polyamides responsible for cell division and lignins [66]. With their amino acid composition, linseed extracts may modulate the synthesis of various chemicals vital to plant growth, such as nucleotides, chlorophyll, hormones, and secondary metabolites. Transcriptomic and metabolomic analyses performed by Nunes-Nesi et al. [68] have uncovered possible regulatory interactions between the metabolism of nitrogen, carbon, sulfur, and phosphorus and primary and secondary metabolism, as well as the systemic response to an added source of amino acids [69]. This may explain the potential mechanisms behind the mode of action of the novel plant biostimulants derived from the linseed extracts. The observed improvements in the soybean yield and changes in the biometric parameters of plants may have resulted from the action of the amino acid pool and the activity of amino acid transporters in the biostimulants, which modulate plant metabolism [70]. Initial results of genetic studies have found that a set of specific amino acids act as signaling molecules that spur detectable changes in plant growth and yields through the induction of complex cascades and signal transduction, leading to changes in gene expression. However, little is still known about the mechanisms that regulate amino acid levels and amino acid-induced signals [69,71]. As suggested by Bettoni et al. [72], such preparations substantially alter nitrogen metabolism in plants. This hypothesis is corroborated by Ertani et al. [73], who found a link between changes in nitrogen metabolism and increased activity of enzymes, including glutamine synthetase and glutamate synthase, which are activated by NH_4_^+^ availability. Ertani et al. [73] and Shahabivand et al. [74] argue that the observed processes may contribute to higher levels of nitrogen and other organic substances in plants and seeds.

The present study also demonstrated that soybean exhibited high variability in biometric traits, which may have resulted from the application of macerates rich in both micro- and macroelements. This correlation is also reflected in the work of Briglia et al. [75], who investigated the potential of using novel biostimulants consisting of a mixture of seaweed and plant extracts formulated with selected micronutrients (Mn, Zn, Mo) to grow corn and soybean. Briglia et al. [75] argue that changes in the crop physiology and chemical composition can be explained through an analysis of molecular mechanisms. The study uncovered a potential mode of action for the biostimulant (consisting of a mixture of seaweed and plant extracts with micronutrients). It was found that this biostimulant formulation upregulated the expression of genes responsible for specific processes in plants, such as nitrogen and phosphate assimilation and metabolism, maltose biosynthesis, carbohydrate transport, and hormone metabolism. In the case of soybean, changes were noted in nitrogen metabolism, increased metal ion transport (including zinc and iron), reduced sulfate levels, and enhanced amino acid biosynthesis [75]. Similar findings were reported by Barone et al. [76], who investigated the molecular-level response of sugar beet to the application of a humic substance-based biostimulant rich in carbon, nitrogen, sulfur, and other elements (K, P, Ca, Mg, Na, Fe, Al). An Open Array Real Time PCR analysis showed that the biostimulant treatment upregulated 53 genes involved in governing metabolic pathways and hormone metabolism in plants, including genes related to sulfur metabolism and phosphate transport, as well as genes involved in cell organization [76].

Research conducted by Godlewska et al. [77] has also demonstrated the viability of plant extracts as biostimulants. These authors used foliar application of plant extracts and reported improvements in the yields and quality parameters of celery leaves and roots. Godlewska et al. [78] pointed to the micro- and macroelement (particularly potassium) content of the extracts as a possible explanation. While the physiological function of potassium has not been fully established, it is known that it is a vital and key element in cellular metabolic processes, protein biosynthesis, assimilate transport, and osmoregulation in cells and stomata [78,79]. Rouphael et al. [26] have found that the nutritional and mineral status of crops treated with biostimulants (including plant extracts) suggests that this mechanism may stem from an increased “nutrient acquisition response”. The presence of signaling molecules, such as amino acids, soluble peptides, microelements, and macroelements, may underlie the observed behavior [80,81]. Genetic analyses have also shown that treatment with biostimulant products increases the expression of genes encoding macronutrient transporters in cell membranes [26,82,83]. Although scarce, the research indicates that plant extracts can be utilized as effective biostimulants, providing far better results than synthetic agrochemicals. Sharifi [84] attributes this superior performance to the mineral content in plant extracts. Zn levels are of particular importance, as zinc plays a crucial role in chlorophyll biosynthesis, lipid and protein metabolism, carbohydrate synthesis, and enzymatic activity [85]. The low solubility of Zn in soils means that crops susceptible to Zn deficiency may suffer from disruption of these physiological and metabolic processes. Accordingly, foliar spraying of crops with plant extracts containing even a small amount of micro- and macroelements (especially zinc) significantly increases yields [84,86], as observed in the present study, in which the linseed extract was used as a biostimulant.

In addition to amino acids, the tested linseed extracts contained fatty acids, minerals, and carbohydrates. These compounds, therefore, may have had a synergistic effect on the analyzed yield and biometric parameters of soybean. The accumulation of unsaturated fatty acids is inherently linked to membrane fluidity and plant adaptation to environmental stress. The present study demonstrated that the application of the linseed-derived biostimulants containing 19 FFAs improved the soybean yield and physiological characteristics. This is a particularly interesting finding, mostly due to the fact that these acids form part of cell membranes, modulate glycerolipids, and serve as a form of carbon and energy storage in triacylglycerols, simultaneously acting as precursors for a variety of bioactive compounds. In addition, their increased levels as an effect of exogenous application in plants indicate improved functioning of the defense system against biotic and abiotic stresses [87]. This is in agreement with the results reported by Godlewska et al. [77,88], who noted that the application of plant extracts induced a change in the fatty acid profile in cabbage and celery root crops [79]. A study on the application of garlic extracts in pepper cultivation conducted by Hayat et al. [28] demonstrated that the extracts mitigated lipid peroxidation and thus prevented the formation of oxidation products inhibiting the activity of membrane enzymes and transport proteins. Still, more research is needed to shed light on the potential modes of action of plant biostimulants in terms of modifying fatty acid levels in arable crops.

Our examination of the use of the biostimulant in soybean cultivation concluded with an economic analysis of the income generated from the production, which is a deciding factor for farmers choosing a product. The study demonstrated that the linseed extracts were effective in improving yields, which was directly reflected in increased returns in actual field conditions. This is corroborated by our previous study on the influence of treatment with *Artemisia absinthium-*derived plant biostimulants on the income from soybean cultivation. The analysis of the economic returns, yields, and costs of treatment in a three-year field trial showed that the foliar administration of *Artemisia absinthium* infusions and macerates led to the highest increases in soybean yields, which in turn ensured stable profits for the producers [4]. These gains are admittedly not spectacular when compared with the economic performance of commercial biostimulants applied to bean crops. Treatment of bean crops with Kelpak SL increased the average income by more than EUR 600 per hectare, whereas the same parameter amounted to over EUR 300 for Terra Sorb [51]. While it is true that this analysis involved a different crop, it still must be admitted that further work is needed to improve the performance of the novel biostimulants. The solution may lie in changing the method of administration or extraction. Nevertheless, the established economic benefits are sufficient to qualify the extracts examined herein as biostimulant products.

The analysis of the physical properties of the novel bioproduct has shown that it has a significantly lower average value of surface tension compared to water and a mixture of water and seaweed concentrate (Kelpak SL). Surface tension can be one of the determinants of the quality of the produced spray droplets which, in turn, affects the degree of coverage on the plant surface. Yu et al. [89] and Xu et al. [90] have examined the influence of surface tension on the drop spread and reported that, besides other factors that influence the spread, e.g., the plant species, leaf surface structure, concentration and type of adjuvant, reduction of surface tension can increase the wetted area. As indicated by the authors of the studies [91,92], reduction of the surface tension of the spray agent may have a positive effect on changing the behavior of droplets on the leaf surface, which may improve the uniformity of spray coverage and spray application efficiency.

## 5. Conclusions

The study has successfully confirmed that the plant biostimulant derived from *L. usitatissimum* seeds can be used as a viable agronomic tool for growing soybean. It can be claimed that the designed and produced biostimulant is rich in bioactive compounds, including amino acids, free fatty acids, carbohydrates, and micro- and macro-elements. The tested *L. usitatissimum* biostimulant showed significantly lower values of surface tension in relation to water and the commercial biostimulant. Thus, further studies are needed to confirm that the reduced surface tension of the biostimulating spray mixture will lead to improved droplet distribution, retention, adhesion to the leaf surface, and consequently leaf wettability during agronomic treatment.

The soybean crops responded well to the foliar application of the preparation, showing improvements at the agronomic and morphological levels. The biometric and agronomic analyses showed that the growth and yields of soybeans improved after the administration of the examined biostimulant. The linseed macerates were effective in terms of soybean yields and profitability. Our findings serve as preliminary evidence for the viability of designing and developing novel biostimulants derived from plants that have been used in other sectors and in traditional medicine. This comprehensive approach in designing and formulating novel bioproducts requires more extensive and targeted research to fully explain the mechanisms behind the improvements observed in the soybean cultivation.

## Figures and Tables

**Table 1:** Multielemental composition of water extract from *L. usitatissimum* (average ± SD).

Element	Extract Obtained by Cold-Soaking
Macroelements (mg·L^−1^)	
Ca	68.737 ± 0.217
K	182.800 ± 1.135
Mg	32.863 ± 0.442
Na	28.380 ± 0.101
P	25.70 ± 0.011
S	33.387 ± 0.061
Microelements (mg·L^−1^)	
Cu	0.239 ± 0.001
Mn	0.069 ± 0.002
Sr	1.185 ± 0.005
Zn	0.399 ± 0.002
Fe	0.033 ± 0.003
Al	0.004 ± 0.003
Toxic metals (mg·L^−1^)	
As	<LLD
Cd	<LLD
Pb	<LLD

<LLD—below limit of detection.

**Table 2:** Carbohydrates and amino acids in water extract from *L. usitatissimum* (average ± SD).

Compound	Extract Obtained by Cold-Soaking
Carbohydrates (mg·mL^−1^)	
Sucrose	1.070 ± 0.021
Glucose	0.540 ± 0.018
Fructose	0.374 ± 0.014
Amino acids (mg·L^−1^)	
Aspartic acid (Asp)	0.661 ± 0.018
Threonine (Thr)	0.214 ± 0.016
Serine (Ser)	0.309 ± 0.019
Glutamic acid (Glu)	1.490 ± 0.015
Proline (Pro)	0.119 ± 0.007
Glycine (Gly)	0.402 ± 0.012
Alanine (Ala)	0.321 ± 0.009
Valine (Val)	0.284 ± 0.010
Isoleucine (Ile)	0.239 ± 0.011
Leucine (Leu)	0.354 ± 0.010
Tyrosine (Tyr)	0.135 ± 0.009
Phenylalanine (Phe)	0.291 ± 0.007
Histidine (His)	0.129 ± 0.012
Lysine (Lys)	0.271 ± 0.014
Arginine (Arg)	0.650 ± 0.015

**Table 3:** Fatty acids in water extracts from *L. usitatissimum* (average ± SD).

Compound	Extract Obtained by Cold-Soaking
Fatty acids (g·100 g^−1^)	
C6:0	0.0001 ± 0.0000
C8:0	0.0006 ± 0.0001
C10:0	0.0007 ± 0.0001
C12:0	0.0011 ± 0.0001
C14:0	0.0032 ± 0.0001
C14:1n5	0.0001 ± 0.0000
C15:0	0.0003 ± 0.0000
C16:0	0.0180 ± 0.0009
C16:1n7	0.0003 ± 0.0000
C17:0	0.0003 ± 0.0000
C18:0	0.0086 ± 0.0005
C18:1n9c + C18:1n9t	0.0217 ± 0.0011
C18:2n6c + C18:2n6t	0.0031 ± 0.0002
C18:3n3 (alpha)	0.0026 ± 0.0002
C20:0	0.0003 ± 0.0000
C22:0	0.0004 ± 0.0000
C23:0	0.0001 ± 0.0000
C24:0	0.0002 ± 0.0000
SFA	0.0342 ± 0.0012
MUFA	0.0221 ± 0.0012
PUFA	0.0057 ± 0.0006
OMEGA 3	0.0026 ± 0.0002
OMEGA 6	0.0031 ± 0.0002
OMEGA 9	0.0217 ± 0.0011

The result (g·100 g^−1^) shows the content of individual fatty acids per 100 g of the tested sample.

**Table 4:** Effect of treatment with *L. usitatissimum* water extract on biometric traits and yield of soybean (average ± SD).

Year	Treatment	Plant Height (cm)	Height of the Location of the First Pod (cm)	Number of Pods (per Plant)	Seed Yield (g m^−2^)	1000 Seed Weight (g)
2017	Control	110.1 ± 4.2 a	10.8 ± 2.4 a	19.4 ± 1.1 a	246.2 ± 13.0 a	177.47 ± 0.89 a
	Cold-soaking	115.3 ± 3.6 a	11.0 ± 1.9 a	22.0 ± 2.2 a	284.9 ± 55.3 a	175.01 ± 2.03 a
2018	Control	107.3 ± 2.6 b	13.4 ± 1.4 b	17.5 ± 1.3 b	236.3 ± 21.5 b	177.58 ± 0.93 a
	Cold-soaking	113.3 ± 2.7 a	11.7 ± 1.2 b	21.9 ± 2.4 a	285.8 ± 24.9 a	174.12 ± 0.86 b
2019	Control	113.1 ± 2.7 a	13.9 ± 2.1 a	17.2 ± 1.5 b	232.1 ± 31.8 b	178.92 ± 0.82 a
	Cold-soaking	118.3 ± 5.8 a	7.5 ± 1.4 b	22.6 ± 3.6 a	302.3 ± 35.7 a	174.82 ± 1.34 b
Average 2017–2019	Control	106.8 ± 3.8 b	12.7 ± 2.3 a	18.0 ± 1.4 b	238.2 ± 23.3 b	177.99 ± 1.05 a
	Cold-soaking	115.6 ± 4.4 a	10.1 ± 2.4 b	22.2 ± 2.6 a	291.0 ± 38.5 a	174.65 ± 1.41 b

Means of the values of selected traits in the columns and years followed by different letters are significantly different at *p* < 0.05.

**Table 5:** Physical properties of the biostimulant from *L. usitatissimum* seeds.

Preparation	Surface Tension (mN m^−1^)	Area (mm^2^)	Volume (µL)
CS	50.82 ± 0.97b	33.69 ± 1.31b	20.15 ± 0.81b
W	71.56 ± 0.71a	43.54 ± 0.69a	28.91 ± 0.54a
SE	71.23 ± 0.74a	43.97 ± 0.86a	29.09 ± 0.66a

CS—cold-soaked *L. usitatissimum* seeds; W—water; SE—seaweed (Kelpak SL); Means of the values of selected traits in the columns followed by different letters are significantly different at *p* < 0.05.

## Data Availability

Data is contained within the article.
